# Host habitat assessment by a parasitoid using fungal volatiles

**DOI:** 10.1186/1742-9994-4-3

**Published:** 2007-02-06

**Authors:** Sven Steiner, Daniel Erdmann, Johannes LM Steidle, Joachim Ruther

**Affiliations:** 1 Institut für Biologie, Freie Universität Berlin, Haderslebener Str. 9, 12163 Berlin, Germany; 2 Tierökologie 220c, Universität Hohenheim, 70593 Stuttgart, Germany

## Abstract

**Background:**

The preference – performance hypothesis predicts that oviposition preference of insects should correlate with host suitability for offspring development. Therefore, insect females have to be able to assess not only the quality of a given host but also the environmental conditions of the respective host habitat. Chemical cues are a major source of information used by insects for this purpose. Primary infestation of stored grain by stored product pests often favors the intense growth of mold. This can lead to distinct sites of extreme environmental conditions (hot-spots) with increased insect mortality. We studied the influence of mold on chemical orientation, host recognition, and fitness of *Lariophagus distinguendus*, a parasitoid of beetle larvae developing in stored grain.

**Results:**

Volatiles of wheat infested by *Aspergillus sydowii *and *A. versicolor *repelled female parasitoids in an olfactometer. Foraging *L. distinguendus *females are known to be strongly attracted to the odor of larval host feces from the granary weevil *Sitophilus granarius*, which may adhere in remarkable amounts to the surface of the grains. Feces from moldy weevil cultures elicited neutral responses but parasitoids clearly avoided moldy feces when non-moldy feces were offered simultaneously. The common fungal volatile 1-octen-3-ol was the major component of the odor of larval feces from moldy weevil cultures and repelled female parasitoids at naturally occurring doses. In bioassays investigating host recognition behavior of *L. distinguendus*, females spent less time on grains containing hosts from moldy weevil cultures and showed less drumming and drilling behavior than on non-moldy controls. *L. distinguendus *had a clearly reduced fitness on hosts from moldy weevil cultures.

**Conclusion:**

We conclude that *L. distinguendus *females use 1-octen-3-ol for host habitat assessment to avoid negative fitness consequences due to secondary mold infestation of host patches. The female response to fungal volatiles is innate, suggesting that host-associated fungi played a crucial role in the evolution of host finding strategies of *L. distinguendus*. Research on the role of host-associated microorganisms in the chemically mediated orientation of parasitoids is still at the beginning. We expect an increasing recognition of this issue in the future.

## Background

The reproductive success of insects is determined not only by the number of eggs females lay but also by the survival and fecundity of their offspring. Therefore, oviposition preference of insects has been predicted to correlate with host suitability for offspring development (preference – performance hypothesis [1]). This hypothesis which has been referred to in the literature as the 'mother knows best' principle [2] has originally been developed for herbivorous insects but is assumed to play an important role also in parasitic Hymenoptera [3]. In parasitoids, host organisms are the only source of nutrients for immature stages [4], and thus, parental fitness particularly depends on accurate assessment of the host sites in terms of their potential to sustain the development of their larvae. Thus, adaptation to reliable cues enabling the evaluation of the host patch quality is a selective advantage for females searching for oviposition sites [5].

Successful parasitization requires commonly a series of successive steps with host habitat location, host location, and host recognition being the major elements [6-8]. Apart from physical cues [9], volatile and non-volatile chemicals have been found to be of considerable importance at almost every level of this foraging process [6,10]. Compounds involved may emanate from the host, host by-products, host food plants, or organisms closely associated with the host [11-13].

Chemical cues can not only attract but also deter parasitoids from entering host sites or from laying eggs into unsuitable hosts. Foraging decisions of insects are affected by both extrinsic factors like the suitability of resources, presence of natural enemies or competitors, and by intrinsic factors like experience and age of the ovipositing female [10,14-17]. In contrast, little attention has been paid to the investigation of chemicals that reliably indicate unfavorable environmental conditions (e.g., temperature, humidity, CO_2 _concentration) within the host habitat [3].

The solitary ectoparasitoid *Lariophagus distinguendus *Förster (Hymenoptera: Pteromalidae) parasitizes immature stages of several stored-product infesting beetle species that develop inside grains and seeds [18,19]. The behavior and chemical cues involved in the host finding process of this species have been extensively examined [20-27]. Volatiles emitted by the larval feces of the granary weevil *Sitophilus granarius *L. and other hosts mediate long-range orientation of the parasitoid during host habitat location [20,25]. Once within the host habitat, females search for reliable cues indicating the presence of their hosts. Again, compounds originating from the larval feces have been shown be crucial for host recognition [24]. On grains infested with the host, *L. distinguendus *females perform a stereotypic sequence of behavioral elements including series of intense antennal drumming, tapping with the abdominal tip on the surface of the infested grain, and finally drilling with the ovipositor into the grain [22]. After immobilizing the host by injecting paralyzing substances, females of *L. distinguendus *typically lay one single egg onto the surface of the host and the hatching larva develops inside the grain while feeding upon the host [28].

Since *L. distinguendus *attacks beetles that often lay their eggs in clumps within stored grain, intense infestation of the grain by a larger number of beetles can lead to a zoned increase of temperature and humidity within the microhabitat [29]. These abiotic conditions promote the invasion of astigmatid mites and particularly the growth of mold [30,31]. At a critical level of secondary infestation, however, environmental parameters in these hot-spots deteriorate and cause an increased insect mortality within the patch. Thus, reproductive success of parasitoids attacking host larvae in areas of high secondary infestation should be lower than in areas of light or no secondary infestation. The ability to detect and avoid these suboptimal oviposition areas would therefore improve the fitness of *L. distinguendus*.

Fungi are well-known to release a multitude of volatile organic compounds originating from different metabolic pathways. The composition of these volatile profiles depends on the fungal species as well as on the growing medium and abiotic factors [32]. However, a variety of chemicals is shared by several species and is reliably released under various conditions. Among these widespread fungal volatiles is a group of aliphatic eight-carbon compounds including 1-octen-3-ol, 3-octanone, and 3-octanol [33] that are derived from enzymatic degradation of unsaturated fatty acids [34]. In most species 1-octen-3-ol occurs as major component, and it has been suggested to use this chemical for the detection of mold infestation in stored grain as well as in living and working environments [35-38].

In the present study, we investigated whether mold infestation of *S. granarius *cultures affects chemical orientation, host recognition behavior, and fitness of *L. distinguendus*. Mold species studied were *Aspergillus sydowii *(Bainier and Sartory) Thom and Church and *A. versicolor *(Vuillemin) Tiraboschi. Furthermore, we determined the amounts of typical eight-carbon fungal volatiles in the weevil cultures and tested the response of female parasitoids to synthetic 1-octen-3-ol. The possible function of fungal volatiles as indicators of suboptimal host patches is discussed.

## Results

### Influence of mold infestation on host finding behavior

**Figure 1:**
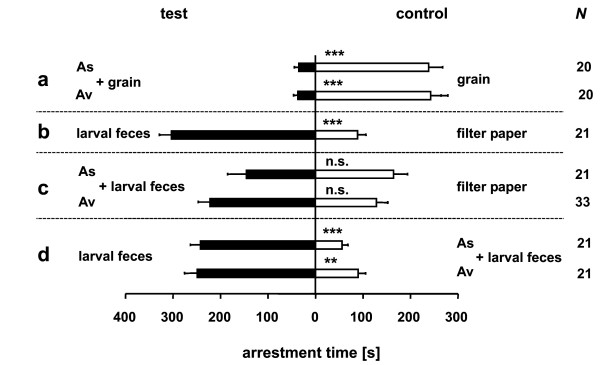
**Response of *L. distinguendus *to moldy grain and host feces**. Mean arrestment time (± SE) of *L. distinguendus *females in the odor fields above test and control chamber of a two choice olfactometer during a 10 min observation period. N.s. = not significant; asterisks indicate significant differences at *P *< 0.01 (**) or *P *< 0.001 (***) (generalized linear model).

*L. distinguendus *females avoided the odor of moldy wheat in an four-chamber olfactometer irrespective of the *Aspergillus *species used to inoculate the grain. In both *Aspergillus *species the odor of uninfested wheat was significantly preferred over inoculated grain (Figure 1a). Parasitoids were strongly arrested by the odor of larval feces from weevil cultures without mold (Figure 1b). However, larval feces from moldy weevil cultures were no longer preferred by the parasitoids when tested against filter paper as a control. The odor of feces from both the *A. sydowii *and *A. versicolor *cultures was neither significantly preferred nor avoided (*A. sydowii*: *P *= 0.76, *A. versicolor*: *P *= 0.058) (Figure 1c). However, when feces from moldy and non-moldy cultures were offered simultaneously, females clearly avoided the feces from the moldy cultures in both cases (Figure 1d).

### Influence of mold infestation on host recognition behavior

**Figure 2:**
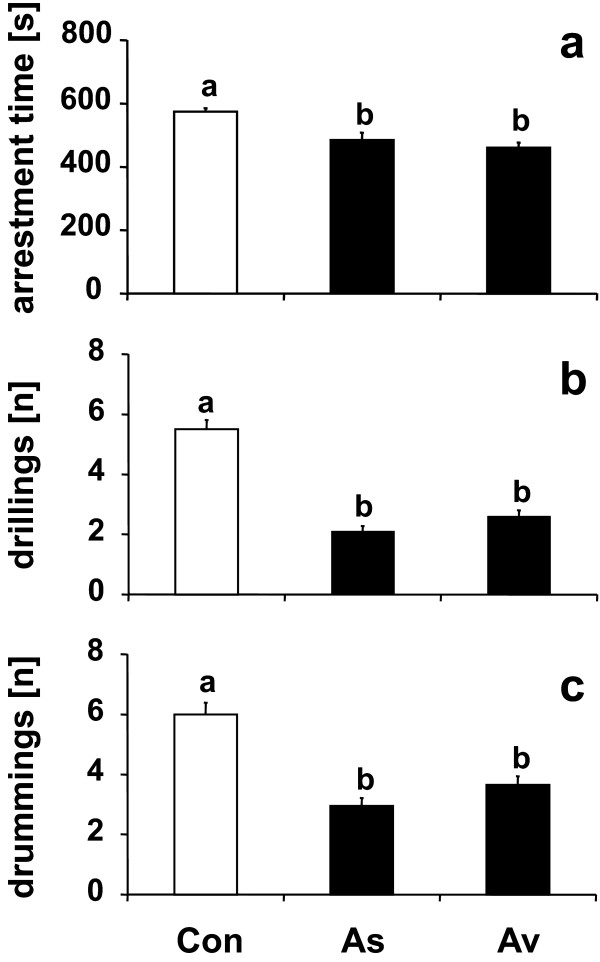
**Host recognition behavior of *L. distinguendus *towards hosts from moldy and non-moldy weevil cultures**. Response of *L. distinguendus *females to host-infested grains originating from non-moldy weevil cultures (Con) and those infested by *A. sydowii *(As) or *A. versicolor *(Av). **(a) **Mean arrestment time on the grain (± SE), **(b) **number of drumming series (± SE), and **(c) **number of drillings (± SE) during a 10 min observation period. Bars with different lowercase letters are significantly different at *P *< 0.001 (Kruskal-Wallis *H*-test followed by Bonferroni-corrected Whitney-Mann *U *tests for multiple comparisons; *N *= 20).

Arrestment time of female parasitoids was significantly decreased on wheat grains from *A. sydowii*- and *A. versicolor*-infested weevil cultures when compared to the non-moldy control grains (Figure 2a). Moreover, characteristic elements of the host recognition behavior (drumming and drilling) were shown less often on grains from the moldy weevil cultures than on control grains (Figure 2b–c). No significant differences were found between grains from weevil cultures infested by *A. sydowii *and *A. versicolor*, respectively (arrestment time: *U *= 145.5, *P *= 0.14; drilling: *U *= 153, *P *= 0.20; drumming: *U *= 138, *P *= 0.09).

### Influence of mold infestation on parasitoid fitness

**Figure 3:**
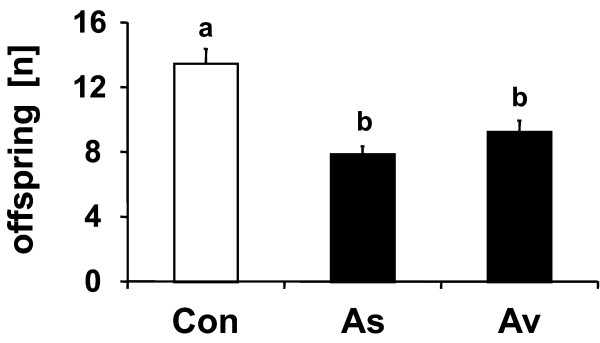
**Fecundity of *L. distinguendus *females on hosts from moldy and non-moldy weevil cultures**. Mean number of offspring (± SE) of *L. distinguendus *females that parasitized host-infested grains from non-moldy weevil cultures (Con) and those infested by *A. sydowii *(As) or *A. versicolor *(Av). Bars with different lowercase letters are significantly different at *P *< 0.01 (one-way ANOVA; *N *= 24–26).

Females of *L. distinguendus *parasitized hosts from both moldy and non-moldy weevil-cultures. However, the number of offspring was significantly lower when host larvae originated from moldy weevil cultures infested with either *A. sydowii *or *A. versicolor *as compared to non-moldy cultures (Figure 3). There was no significant difference in the number of offspring between the two mold species (*P *= 0.43). Body size as measured by the length of the tibiae was significantly affected by the infestation with mold. Parasitoids that had developed on hosts from non-moldy weevil cultures had longer tibiae (female: 0.579 mm ± 0.003; male: 0.5058 mm ± 0.005) than individuals from *A. sydowii*- (female: 0.571 mm ± 0.003; male: 0.5058 mm ± 0.003) or *A. versicolor*-moldy cultures (female: 0.573 mm ± 0.003; male: 0.500 mm ± 0.003). Moreover, females of *L. distinguendus *had generally longer tibiae than males. There was no interaction observed between treatment and sex (Table 1).

**Table 1:** Statistical analysis of tibia lengths of male and female offspring

**Source**	***df***	***MS***	***F***	***P***
Treatment (Con, As, Av)	2	0.5429	722.706	**< 0.001**
Sex (m, f)	1	0.0030	3.950	**0.020**
Treatment × Sex	2	0.0001	0.154	0.857
Error	391	0.00075		

Two-way ANOVA of tibia lengths of *L. distinguendus *males (m) and females (f) developing in non-moldy weevil cultures (Con) and weevil cultures infested by *A. sydowii *(As) or *A. versicolor *(Av) (*df: *degrees of freedom; *MS*: mean squares).

### Volatile analysis of feces from moldy and non-moldy weevil cultures

Fecal volatiles from moldy and non moldy weevil cultures were collected by closed loop stripping and analyzed by coupled gas chromatography-mass spectrometry (GC-MS). The major eight-carbon fungal volatile detected in larval feces from moldy weevil cultures was 1-octen-3-ol accompanied by lower amounts of 3-octanone, and 3-octanol (Table 2). In larval feces from non-moldy host cultures all compounds were found only in traces. The headspace of *A. sydowii *contained additionally some sesquiterpenes as minor compounds.

**Table 2:** Amounts of typical eight-carbon fungal volatiles in larval host feces

**Compound**	**Con**	**As**	**Av**
1-octen-3-ol	17 ± 4	516 ± 5	730 ± 46
3-octanone	19 ± 4	116 ± 14	272 ± 47
3-octanol	5 ± 1	14 ± 2	120 ± 7

Mean amounts (ng per sampling ± SE, *N *= 3) of typical eight-carbon fungal volatiles from the headspace of larval feces from non-moldy weevil cultures (Con) and weevil cultures infested by *A. sydowii *(As) or *A. versicolor *(Av).

### Response to synthetic fungal volatile

**Figure 4:**
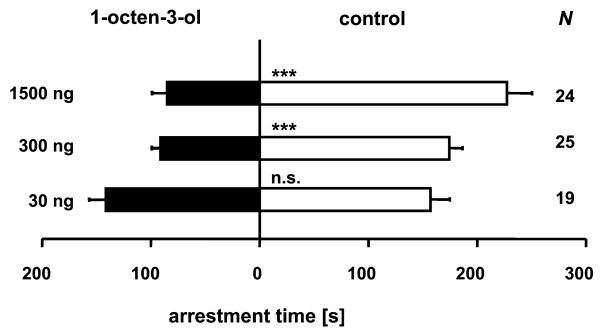
**Response of *L. distinguendus *to synthetic 1-octen-3-ol**. Mean arrestment time (± SE) of *L. distinguendus *females in the odor fields above test and control chamber of a two choice olfactometer during a 10 min observation period. Test fields were treated with synthetic 1-octen-3-ol at different doses; control fields were treated with pure solvent. N.s. = not significant; *** indicates significant differences at *P *< 0.001 (generalized linear model).

*L. distinguendus *females avoided significantly synthetic 1-octen-3-ol at doses between 1500 to 300 ng (Figure 4). At a dose of 30 ng, however, parasitoids were neither repelled nor attracted to the synthetic chemical (*P *= 0.62).

## Discussion

To study the ability of parasitoids to use chemical cues for the assessment of host habitat quality, the response of *L. distinguendus *was tested to grains and host feces infested with the two mold species *A. sydowii *or *A. versicolor*. Both species co-occur with the parasitoid's hosts in its natural stored grain habitat under humid and warm conditions. The olfactometer experiments clearly demonstrated that naïve females of *L. distinguendus *avoid the odor of grains infested by either mold species when offered simultaneously with healthy grains. Likewise, odor from moldy host feces was avoided when non-moldy host feces were offered as an alternative. Obviously, *A. sydowii *and *A. versicolor *release volatiles that are repellent for females of *L. distinguendus*. As shown in experiments in which non-moldy and moldy feces were tested against filter paper only, the repellent effect of volatiles released by both mold species was even strong enough to neutralize the attractive effect of the odor released by non-moldy host feces. In agreement with these results, host recognition behavior of wasps was much more intense on weevil-infested grains from non-moldy cultures when compared to those from moldy ones. Thus, in a bulk of weevil-infested stored grain containing patches with and without mold, parasitoids would most likely orientate towards non-moldy areas during the host finding process and prefer hosts in non-moldy grains for oviposition.

Chemical analyses and further olfactometer tests pointed to 1-octen-3-ol as a chemical cue that might be responsible for the avoidance response of the parasitoid. This compound was emitted from moldy feces in amounts that repelled *L. distinguendus *females when applied as synthetic chemical to filter paper. Lower concentrations of 1-octen-3-ol, comparable to the amounts released by feces from cultures with no visible mold, had no effect on the wasps. Therefore, results of experiments using feces and synthetic 1-octen-3-ol match very well provided that release rates were comparable. However, also the other eight-carbon volatiles and further compounds may have contributed to the avoidance response of the parasitoids. Interestingly, 1-octen-3-ol is released as a major volatile not only by the two *Aspergillus *species studied here but also by numerous fungi from other taxa [32,33,37,39,40]. Thus, 1-octen-3-ol reliably indicates fungal growth in stored grain.

The ultimate reason for the avoidance of fungal odor by the parasitoid is probably a reduced fitness, as shown here by a lower number of offspring on hosts from moldy weevil cultures. This might be due to one or several of the following mechanisms: (1) Female parasitoids laid a reduced number of eggs on hosts from moldy cultures. This hypothesis is supported by the fact that important elements of the host recognition behavior (arrestment, drumming, and drilling) were shown to a significantly lesser extend when host-infested grains from moldy weevil cultures were offered. Thus, moldy grains might be less attractive for oviposition by *L. distinguendus*. (2) Parasitoids had a higher pre-emergence mortality due to mycotoxins. Numerous mold species including the genus *Aspergillus *are known to synthesize toxic metabolites [41,42]. These might have decreased the parasitoid's and/or the host's survival rate. (3) Competition between mold and host larvae for wheat nutrients decreased host performance allowing a lower number of parasitoid larvae to develop into adults. It is known from many studies that parasitoid larvae developing on suboptimal hosts may be subjected to an increased pre-emergence mortality as well as a reduced longevity or fecundity [[3] and references therein]. Parasitoids from moldy weevil cultures were significantly smaller supporting the hypothesis that the hosts were not of equal quality. A correlation between body size and the reproductive success has been reported for *L. distinguendus *[43]. Larger females were shown to produce more offspring than smaller individuals because they lived longer and were more fertile. In contrast, size was less important for males. Thus, mold infestation might also influence the number of offspring in the second generation resulting in a decreased inclusive fitness. However, it has to be tested whether the subtle differences in body size observed in this study can actually influence fitness of the parasitoids. (4) Secondary infestation by *Aspergillus *mold caused unfavorable environmental conditions for parasitoid and/or host development. Local mass breeding of insects in stored grain and subsequent mold infestation can lead to distinct areas with extreme abiotic conditions. The development of such hot-spots has been described in detail [29]. Initially, the metabolic activity of the primary pest leads to a moderate increase of temperature and humidity providing optimal conditions for moisture-sensitive secondary pests like astigmatid mites and mold. With increasing growth of fungi, however, moisture and temperature as well as the concentration of carbon dioxide may reach levels that cause a decline of insect populations and finally a breakdown of the hot-spot [29]. *L. distinguendus *and some of its hosts clearly prefer lower humidities over those commonly found in hot spots [44,45]. Parasitoid females may recognize at an early stage the development of such unfavorable environmental conditions by avoiding fungal volatiles like 1-octen-3-ol. Thereby, they reduce negative fitness consequences resulting from secondary mold infestation of host patches. Nevertheless, parasitoids are able to develop in hosts from moldy weevil cultures. Therefore, it makes sense that there was a tendency of females to prefer the odor of larval feces from moldy weevil cultures (*A. versicolor*) in absence of the more attractive alternative, i.e., feces from non-moldy host cultures. The fact that the response of females to the fungal volatile is innate, suggests that host-associated fungi played a crucial role in the evolution of host finding strategies in *L. distinguendus*.

## Conclusion

The present study is a good example for the 'mother knows best' principle [2] in parasitoids demonstrating that predictions of the preference – performance hypothesis originally made for phytophagous insects may also apply for carnivores. Furthermore, the study is one of the very few demonstrating that infochemicals used by parasitoids for host habitat assessment are produced by host-associated microorganisms. Hitherto, host associated bacteria [46,47] or fungi [48] have only been shown to produce kairomones that attract parasitoids to their hosts rather than helping them to avoid suboptimal host patches.

Current research on plant-herbivore systems discovers more and more the important role of plant pathogens that influence larval performance and oviposition decisions [2,49-51]. However, only few studies have addressed the question whether plant pathogens may also influence the pattern of herbivore-induced plant volatiles and thereby trigger the response of parasitoids in tritrophic interactions [52,53]. Research on the direct or indirect role of microorganisms in the chemically mediated orientation of parasitoids is still at the beginning. We expect an increasing recognition of this issue in the future.

## Methods

### Insect cultures

Insect cultures were kept at constant conditions of 25°C, 65% relative humidity (RH), and a daily light:dark cycle of 16:8 h. The *L. distinguendus *strain used in the experiments was collected on *Stegobium paniceum *L. in a flour mill in Uzwill, Switzerland. In our laboratory the parasitoid was reared on larvae (3^rd ^and 4^th ^instar) of the granary weevil *Sitophilus granarius *in wheat grains (*Triticum aestivum *L., var. Batis), as described elsewhere [20].

### Insects for experiments

For all experiments except for experiment 2, naïve *L. distinguendus *females were used, i.e., individuals without mating and oviposition experience. For this purpose, parasitoids were collected immediately after emergence and held in groups of at most 15 individuals in Petri dishes lined with moistened filter paper. To obtain experienced parasitoids, freshly emerged females were kept together with males in a Petri dish containing host-infested grains for parasitization. After three days, females were removed and kept without hosts in Petri dishes on moistened filter paper. One hour before experiments, *L. distinguendus *were isolated in 1.5 ml microcentrifuge tubes for acclimation at room temperature. Behaviors shown by the parasitoids during experiments were recorded by using the computer software The Observer 3.0 (Noldus, Wageningen, The Netherlands).

### Fungal cultures

The mold species *A. versicolor *(Strain no.: BBA 72388) and *A. sydowii *(Strain no.: BBA 72389) used in the experiments were obtained from the culture collection of the Biologische Bundesanstalt für Land- und Forstwirtschaft, Institute of Plant Virology, Microbiology, and Biosafety, Berlin, Germany. The isolates originated from intense weevil- and fungi-infested grain cultures of our institute.

Stock cultures of *A. sydowii *and *A. versicolor *were freeze-dried and stored at 4°C. Subcultures were cultivated on nutrient deficient agar SNA at room temperature for 1 to 2 weeks. A 10 ml portion of sterile water was added to the subculture and the surface was scraped off until conidia were suspended. The suspension was filtered through a folded filter paper (S&S 595, Schleicher & Schuell, Dassel, Germany) and the conidia concentrations of the *Aspergillus *species were determined by counting in a Bürker counting chamber and adjusted with sterilized water to 1.5 × 10^6 ^conidia per ml.

### Preparation of moldy grain and weevil cultures

Three batches of wheat grain (750 g each) were autoclaved for 30 min. Two batches were treated with 45 ml conidia suspension of *A. sydowii *and *A. versicolor*, respectively. For control, the third batch of grain was treated with 45 ml sterilized water. The three batches of grain were incubated separately for four weeks in desiccators at 25°C. The humidity within the desiccators was adjusted to 65% by a saturated solution of ammonium nitrate [54]. After this incubation period, mold was visible in the two treated batches of wheat but not in the control. Adult *S. granarius *(75 ml) were added to each batch of grain, allowed to lay eggs into the grains for seven days and subsequently removed. Larval feces used for the olfactometer bioassays and the chemical analysis were obtained by sieving the grain of treated and control cultures 21 to 28 days after the adults had been removed. After removal of the larval feces, weevil-infested grains of mold-treated and control cultures were used to investigate the influence of mold on the host recognition behavior of *L. distinguendus *in experiment 2 and fitness of the parasitoids in experiment 3.

### Four-chamber olfactometer

The response of *L. distinguendus *females to different odor samples was examined using a static four-chamber olfactometer [20,21]. The olfactometer consisted of a cylinder made of acrylic (4 cm high, ∅ 19 cm) divided into four chambers by crosswise-arranged vertical plates. The top of the cylinder was covered by a plastic gauze (mesh 0.1 mm) functioning as a walking arena for the parasitoids. A lid consisting of a plastic ring (4 mm high, 19 cm ∅) and a glass plate was placed on top of the cylinder to prevent the parasitoids from escaping. Odor samples were placed in one of the chambers (test chamber) using a Petri dish (5.5 cm ∅). The opposite chamber was used as control chamber and the remaining two chambers adjacent to the test chamber were considered as buffer zones. A single female was released into the center of the walking arena and the time the parasitoid spent in the field above the test or control chamber was recorded for 10 minutes. Parasitoids that were motionless more than 50% of the total observation time were assumed to be unmotivated and excluded from statistical analysis. To avoid biased results due to possible side preferences of the parasitoids, the position of the samples and the controls was rotated clockwise after each test. Walking arena and glass plate were regularly cleaned with ethanol and demineralized water. Odor sources were exchanged after five individuals had been tested.

### Experiment 1: Influence of mold infestation on host finding behavior

The following odor samples were offered in the olfactometer: (A) 10 g grain infested with *A. sydowii *or *A. versicolor *vs. 10 g untreated grain (*N *= 20 for each treatment), (B) 200 mg larval feces from the non-moldy control culture vs. a piece of brown filter paper (*N *= 21), (C) 200 mg larval feces from the mold-infested weevil cultures (*A. sydowii*: *N *= 21, *A. versicolor*: *N *= 31) vs. brown filter paper, and (D) 200 mg larval feces from the mold-infested weevil cultures (*A. sydowii*: *N *= 21, *A. versicolor*: *N *= 21) vs. 200 mg larval feces from the non-moldy control culture.

### Experiment 2: Influence of mold infestation on host recognition behavior

The host-recognition behavior of experienced *L. distinguendus *females was examined in a bioassay chamber (10 mm ∅ × 3 mm high) made from acrylic [55]. Single grains from weevil cultures infested with *A. sydowii *or *A. versicolor *or those from the control culture were presented to individual females. Arrestment time on the grains and characteristic elements of the host-recognition behavior (number of drumming series and drilling) were recorded for 10 min using a stereomicroscope under illumination of a microscope light. Grains were exchanged after every parasitoid tested (*N *= 20 for each treatment).

### Experiment 3: Influence of mold infestation on parasitoid fitness

This experiment was done to estimate the offspring production of *L. distinguendus *females in a no-choice situation with hosts from moldy and non-moldy weevil cultures. Virgin females were allowed to copulate with a male and subsequently placed individually in Petri dishes containing weevil-infested grains from the moldy weevil-cultures (*A. sydowii*: *N *= 25, *A. versicolor: N *= 23) or the control culture (*N *= 25). Weevil-infestation of grains was equal in moldy (*A. sydowii*: 82%, *A. versicolor*: 76%) and non-moldy (78%) weevil cultures. Parasitoids were allowed to oviposit until their death. Offspring was assessed by counting the emerged parasitoids after 30 days. In addition, hind tibia length [56,57] was used to measure the body size of randomly selected individuals from the offspring of the three treatments (*A. sydowii: N *= 102, *A. versicolor: N *= 91, control: *N *= 143).

### Experiment 4: Response to synthetic 1-octen-3-ol

The response of *L. distinguendus *females to the typical fungal volatile 1-octen-3-ol (98%, Sigma-Aldrich, Steinheim, Germany) was investigated in another olfactometer experiment. The chemical was applied at different doses [1500 ng (*N *= 24), 300 ng (*N *= 25), and 30 ng (*N *= 19)] to filter paper discs (∅ 4 cm, Melitta, Minden, Germany) and offered in the test chamber of the olfactometer. Paper discs treated with the pure solvent (30 μl dichloromethane) were used as control.

### Volatile collection

Volatiles emitted from the larval feces of the three different weevil-cultures (*N *= 3 for each treatment) were collected by closed loop stripping (CLS) as described elsewhere [21]. Volatiles from 5 g larval feces were collected for 4 h on a 1 mm charcoal layer (5 mg) of a CLS-adsorption tube (65 mm length × 5 mm ∅) (Gränicher & Quartero, Daumazan, France). The volatiles were eluted with 25 μl dichloromethane containing 5 ng/μl methyl undecanoate as an internal standard and used for chemical analysis by GC-MS.

### GC-MS analysis

Volatile extracts were analyzed by GC-MS using a Fisons 8060 GC (Fisons Instruments) equipped with a 30 m × 0.32 mm ID × 0.25 μm film thickness DB-5 ms column (J & W Scientific, Folsom, CA, USA) and coupled to a Fisons MD800 quadrupole MS operated in the electron impact (EI) mode at 70 eV. Helium was used as carrier gas at a head pressure of 10 kPa. The oven temperature was 40°C for 4 min and then rose to 280°C at a rate of 5°C/min. The final temperature was held for 10 min for thermal cleaning of the column. Chemicals were identified by comparison of mass spectra and retention times with those of synthetic reference compounds (Sigma-Aldrich, Steinheim, Germany). For quantification of selected compounds, the peak area of each volatile was related to the peak area of the internal standard.

### Statistical analysis

The arrestment times of female parasitoids spent in test and control field of the olfactometer (experiments 1 and 4) were analyzed by a general estimator equation-generalized linear model (GEE-GLM, SAS version 9.1, PROC GENMOD). Model fitting was carried out with a Poisson error distribution and log link function. Residuals were inspected for normality. Since odor samples in these experiments were renewed after every five insects, we tested not only the factor *treatment *but also the factors *sample *and *order *as explanatory variables for the dependent variables (= arrestment times) to make sure that neither the order of the parasitoids nor the individual sample had a significant influence on their response. However, in none of the experiments a significant effect of order or sample was found indicating that multi-used odor sources elicited constant responses in the parasitoids during the experimental period. The arrestment time on the weevil-infested grains and the number of drumming series and drillings in experiment 2 were analyzed by the Kruskal-Wallis *H*-test followed by multiple Bonferroni-corrected Mann-Whitney *U*-tests for individual comparisons (data partially not normally distributed). Number of offspring (experiment 3) was compared by a one-way ANOVA and subsequent least significant difference (LSD) tests for post hoc comparison. Tibia lengths were analyzed by a two-way ANOVA (factor 1: treatment, factor 2: sex). Statistical analyses except for the GEE-GLM were done using Statistica 4.5 scientific software (StatSoft, Hamburg, Germany).

## Competing interests

The author(s) declare that they have no competing interests.

## Authors' contributions

SS participated in the design of the experiments, performed the chemical analyses and some of the behavioral experiments, and drafted the manuscript. DE performed some of the behavioral and fecundity experiments and did the microbiological part of the study. JLM contributed substantially to the design of the experiments. JR designed the experiments and helped to draft the manuscript. All Authors read and approved the final manuscript.
